# The impact of Ghana's National Health Insurance Scheme median pharmaceutical pricing methodology and reimbursement policy on the pharmaceutical system

**DOI:** 10.1186/2052-3211-8-S1-P5

**Published:** 2015-10-05

**Authors:** Dumebi O Mordi, Kwesi Eghan, Jim Rankin

**Affiliations:** 1 Center for Pharmaceutical Management, Management Sciences for Health, Arlington, Virginia, 22203, USA

## Background

Over 10 years after the establishment of the National Health Insurance Scheme (NHIS) in Ghana, the use of a median pricing methodology for pharmaceuticals remains a topic of debate due to its positive and negative outcomes. Residual effects of this pricing methodology include proliferation of low-quality medicines, irrational medicine use, insurance fraud and other untoward outcomes. Of particular interest is the ripple effect of this median pricing policy on the entire pharmaceutical system. Ghana's pharmaceutical system has limited local production capacity and is heavily dependent on importation. Medicine prices continue to rise and the medicine reimbursement value constitutes an increasingly larger proportion of overall claims values. With finite resources allocated to the NHIS, this presents a sustainability challenge to be addressed promptly.

## Methodology

During a 2015 study of the pricing policy and the system, data were collected using the Management Sciences for Health (MSH) qualitative assessment tool for medicine benefit management programs. The tool was implemented during interviews with pharmaceutical system stakeholders in the Greater Accra, Cape Coast and Kumasi regions of Ghana. Stakeholders from tertiary hospitals, polyclinics, private and public pharmacies, importers/wholesalers, Ministry of Health, professional organizations etc. were interviewed to gather anecdotal evidence about the impact of pricing policy. Quantitative medicine claims data for the period July-December 2014 were also collected to analyze patterns of medicine utilization and reimbursement values. Market dynamics, foreign exchange and medicine prices were considered in the comparison of reimbursement prices with the market value.

## Results

Influential factors including importation fees, foreign exchange, demand and supply chain challenges are not considered in the median pricing methodology. The NHIS pricing methodology is widely viewed as outdated, inefficient and a contributor to delayed reimbursements, subsequent financial crisis and a steady decline in the availability of medicines within the system. Anecdotal and quantitative evidence indicates the critical need for a revised pricing policy to include the key pricing factors in the near future.

## Conslusions

As countries strive towards Universal Health Coverage (UHC), it is critical to consider medicines in all conversations, design and planning of programs due to their clinical and financial impact. Lessons learned from Ghana include the importance of evidence-based pharmaceutical pricing and reimbursement policies, detailed deliberation about medicine benefits during initial UHC designs and policy discussions and system strengthening in support of universal health coverage plans.

